# Optimal Flow Sensing for Schooling Swimmers

**DOI:** 10.3390/biomimetics5010010

**Published:** 2020-03-09

**Authors:** Pascal Weber, Georgios Arampatzis, Guido Novati, Siddhartha Verma, Costas Papadimitriou, Petros Koumoutsakos

**Affiliations:** 1 Computational Science and Engineering Laboratory, ETH Zürich, Clausiusstrasse 33, 8092 Zürich, Switzerlandarampatzis@collegium.ethz.ch (G.A.); novatig@ethz.ch (G.N.); 2 Collegium Helveticum, 8092 Zurich, Switzerland; 3 Department of Ocean and Mechanical Engineering, Florida Atlantic University, Boca Raton, FL 33431, USA; vermas@fau.edu; 4 Harbor Branch Oceanographic Institute, Florida Atlantic University, Fort Pierce, FL 34946, USA; 5 Department of Mechanical Engineering, University of Thessaly, Pedion Areos, GR-38334 Volos, Greece; costasp@uth.gr

**Keywords:** bayesian experimental design, optimal sensor placement, schooling, self-propelled swimmers, lateral line

## Abstract

Fish schooling implies an awareness of the swimmers for their companions. In flow mediated environments, in addition to visual cues, pressure and shear sensors on the fish body are critical for providing quantitative information that assists the quantification of proximity to other fish. Here we examine the distribution of sensors on the surface of an artificial swimmer so that it can optimally identify a leading group of swimmers. We employ Bayesian experimental design coupled with numerical simulations of the two-dimensional Navier Stokes equations for multiple self-propelled swimmers. The follower tracks the school using information from its own surface pressure and shear stress. We demonstrate that the optimal sensor distribution of the follower is qualitatively similar to the distribution of neuromasts on fish. Our results show that it is possible to identify accurately the center of mass and the number of the leading swimmers using surface only information.

## 1. Introduction

Fish navigate in their habitats by processing visual and hydrodynamic cues from their aqueous environment. Such cues may serve to provide awareness of their neighbors as fish adapt their swimming gaits in groups. Early studies have shown that vision is a critical factor for fish schooling [1]. However, more recent studies have shown that even blinded fish can keep station in a school [2]. Such capabilities are of particular importance in flow environments where vision capabilities may be limited [3]. The flow environment is replete with mechanical disturbances (pressure, shear) that can convey information about the sources that generated them. Fish swimming in groups have been found to process such hydrodynamic cues and balance them with social interactions [4,5]. In order to detect mechanical disturbances in terms of surface pressure and shear stresses fish have developed a specialized organ, the lateral line system. The mechanoreceptors in the lateral line—allowing the sensing of the disturbances in water—are called neuromasts. A number of studies and experiments have shown that the functioning of the lateral line is crucial for several tasks [6,7]. Experiments with trout in the vicinity of objects have shown its importance for Kármán gaiting and bow wake swimming as well as energy efficient station keeping [8,9]. Using the information contained in the flow, the cylinder diameter, the flow velocity, and the position relative to the generated Kármán vortex street were quantified [10,11]. Using blind cave fish, several studies have shown the importance of the lateral line to detect the location and the shape of surrounding objects and avoid obstacles [12,13,14,15]. In another study, the feeding behavior of blinded mottled sculpin was tested and it was found that they use their lateral line system to detect prey [16]. It was also found that blind fish manage to keep their position in schools and lose this ability with a disabled lateral line organ [17]. The importance of the lateral line was also shown for enhanced communication [18], the selection of habitats [19] and rheotaxis [20].

In this work, we mimic the mechanosensory receptors, more specifically the sub-surface ‘canal’ neuromasts and superficial neuromasts [21,22]. The neuromast on the fish skin are used to detect shear stresses, where the ones residing in the lateral line canals are used to detect pressure gradients [23,24,25,26,27]. Due to the filtering nature of the canals, the detection of small hydrodynamic stimuli against background noise is improved for the subsurface neuromasts [28].

The effectiveness and versatility of the lateral line organ has yielded several bio-inspired artificial flow sensors [29,30,31,32,33]. Arranging these sensors in arrays on artificial swimmers has attracted attention to transform underwater sensing [3,34,35,36,37,38,39]. Here, leveraging the intelligent distributed sensing inspired by the lateral line showed to be effective in robots moving in aquatic environments [40,41,42,43,44,45].

In order to better use and understand the capabilities of the artificial sensors several studies regarding the information content in the flow and optimal harvesting of this information were performed: The prevalence of information on the position of a vibrating source was shown to be linearly coded in the pressure gradients measured by the subsurface neuromasts [46]. Furthermore, it was shown that the variance of the pressure gradient is correlated with the presence of lateral line canals [47]. In [48], fish robots equipped with distributed pressure sensors for flow sensing were combined with Bayesian filtering in order to estimate the flow speed, the angle of attack, and the foil camber. Other studies have focused on dipole sources in order to develop methods that extract information and optimize the parameters of the sensing devices [49,50]. In a recent study artificial neural networks were employed to classify the environment using flow-only information [51,52,53,54]. In order to find effective sensor positions weight analysis algorithms were employed [55].

Following an earlier work for detection of flow disturbances generated from single obstacles [56], we examine the optimality of the spatial distribution of sensors in a self-propelled swimmer that infers the size and the relative position of the leading school. We combine numerical simulations of the two-dimensional Navier–Stokes equation and Bayesian optimal sensor placement to examine the extraction of flow information by pressure gradients and shear stresses and the optimal positioning of associated sensors. The present work demonstrates the capability of sensing a rather complex system using information of shear and pressure. Such information is available both, to biological organisms and artificial swimmers. We remark that the present work does not aim to reproduce biological systems but rather reveal algorithms that may be applicable to robotic systems. At the same time, we find that the identified optimal sensor locations for the two-dimensional artificial swimmers have similarities to biological systems indicating common governing physical mechanisms for the hydrodynamics of natural and artificial swimmers.

The paper is organised as follows: In Section 2.1 we describe the numerical simulations and in Section 2.2 the process of Bayesian optimal experimental design. We present our results in Section 3 and conclude in Section 4.

## 2. Materials and Methods

### 2.1. Flow Simulations

The swimmers are modeled by slender deforming bodies of length *L* which are characterized by their half-width w(s) along the midline [57,58]

(1) $$w\left(s\right)=\left\{\begin{array}{cc} \sqrt{2w_{h}s-s^{2}}\;, & 0\leq s<s_{b}\;,\\ w_{h}-\left(w_{h}-w_{t}\right)\left(\frac{s-s_{b}}{s_{t}-s_{b}}\right)\;, & s_{b}\leq s<s_{t}\;,\\ w_{t}\frac{L-s}{L-s_{t}}\;, & s_{t}\leq s\leq L\;. \end{array}\right.$$

**Figure 1:**
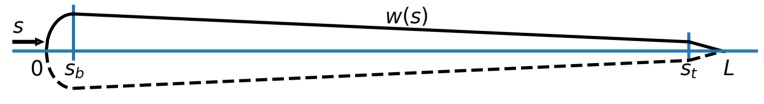
Parametrization of the swimmer surface as described in Equation (1).

A sketch of the parametrization is presented in Figure 1. Following [59], we use $w_{h}=s_{b}=0.04L$, $s_{t}=0.95L$ and $w_{t}=0.01L$. The swimmers propel themselves by performing sinusoidal undulations of their midline. This motion is described by a time dependent parameterization of the curvature,

(2) $$k\left(s,t\right)=A\left(s\right)\sin\left(\frac{2\pi t}{T_{p}}-\frac{2\pi s}{L}\right)\;.$$

Here $T_{p}=1$ is the tail-beat period and *A* is the undulation amplitude which linearly increases from A(0)=0.82/L to A(L)=5.7/L to replicate the anguilliform swimming motion described by [60]. Given the curvature along *s* and a center of mass, the coordinates r(s,t) of the swimmer’s midline can be computed by integrating the Frenet–Serret formulas [59]. In turn, the half-width w(s) and the coordinates r(s,t) characterize the swimmer’s surface.

The flow environment is described by numerical simulations of the two-dimensional incompressible Navier–Stokes equations (NSE) in velocity-pressure (u-*p*) formulation. The NSE are discretized with second order finite differences and integrated in time with explicit Euler time stepping. The fluid-structure interaction is approximated with Brinkman penalization [58,61,62] by extending the fluid velocity u inside the swimmers’ bodies and by including in the NSE a penalization term to enforce no-slip and no-through boundary conditions,

(3) $$\begin{array}{c} \frac{u^{k+1}-u^{k}}{\delta t}=-\nabla p^{k}-\left(u^{k}\cdot \nabla \right)u^{k}+\nu \Delta u^{k}+\sum_{i=1}^{N_{s}}\lambda \chi_{i}\left(u_{s,i}^{k}-u^{k}\right)\;. \end{array}$$

Here, ν is the kinematic viscosity, λ=1/δt is the penalization coefficient, $N_{s}$ is the number of swimmers, $u_{s,i}^{k}$ is the velocity field imposed by swimmer *i* (composed of translational, rotational and undulatory motions), and $\chi_{i}$ is its characteristic function which takes value 1 inside the body of swimmer *i* and value 0 outside. The characteristic function $\chi_{i}$ is computed, given the distance of each grid-point from the surface of swimmer *i*, by a second-order accurate finite difference approximation of a Heaviside function [63]. The pressure field is computed by pressure-projection [58,64],

(4) $$\Delta p^{k}=\frac{1}{\delta t}\nabla \cdot {\tilde{u}}^{k}-\frac{1}{\delta t}\sum_{i=1}^{N_{s}}\chi_{i}\nabla \cdot {\tilde{u}}^{k}\;,$$

where ${\tilde{u}}^{k}=u^{k}-\left(u^{k}\cdot \nabla \right)u^{k}+\nu \Delta u^{k}$. The terms inside the summation in Equation (4) are due to the non-divergence free deformation of the swimmers.

#### 2.1.1. Schooling Formation

The tail-beating motion that propels forward a single swimmer generates in its wake a sequence of vortices. The momentum contained in the flow field induces forces which swimmers in schooling formation must overcome to maintain their positions in the group [65]. In this study, we maintain the schooling formation for multiple swimmers by employing closed-loop parametric controllers. The tail beating frequency $T_{p,i}$ of each swimmer *i* is increased or decreased if it lags behind or surpasses respectively a desired position $\Delta x_{i}$ in the direction of the school’s motion,

(5) $$T_{p,i}=T_{p}\left(1-\Delta x_{i}\right)\;.$$

The mean school trajectory is adjusted by imposing an additional uniform curvature $k_{C,i}$ along each swimmer’s midline in order to minimize its lateral deviation $\Delta y_{i}$ and its angular deflection $\Delta \theta_{i}$,

(6) $$k_{C,i}={\left[\Delta y_{i},\langle \Delta \theta_{i}\rangle \right]}_{–}+{\left[\langle \Delta y_{i}\rangle ,\Delta \theta_{i}\right]}_{–}+{\left[\langle \Delta y_{i}\rangle ,\langle \Delta \theta_{i}\rangle \right]}_{–}\;.$$

Here, 〈·〉 defines an exponential moving average with weight $\delta t/T_{p}$, which approximates the integral term found in PI controllers and

(7) $${\left[a,b\right]}_{–}=\left\{\begin{array}{cc} |a|b\;,\; & \mathrm{if}\;ab<0\;,\\ 0\;,\; & \mathrm{otherwise}\;. \end{array}\right.$$

The formulation in Equation (6) indicates that if both the lateral displacement and the angular deviation are positive (or both negative) the swimmer will gradually revert to its position in the formation. Conversely, if $\Delta y_{i}$ and $\Delta \theta_{i}$ have different signs the displacement has to be corrected by adding (or subtracting) curvature to the swimmer’s midline.

#### 2.1.2. Flow Sensors

We distinguish two types of sensors on the swimmer body. The superficial neuromasts detect flow stresses and the subcanal neuromasts pressure gradients [31,66,67]. From the numerical solution of the 2D Navier–Stokes equation we obtain the flow velocity u=(u,v) and the pressure *p* at every point of the computational grid. The surface values of these quantities are obtained through a bi-linear interpolation from the nearest grid points. We perform offline analysis by recording the interpolated pressure *p* and flow velocity u in the vicinity of the body. We remark that we have neglected points near the end of the body to reduce the influence of large flow gradients that are generated by the motion and sharp geometry of the tail. The shear stresses are computed on the body surface using the local tangential velocity in the two nearest grid points. Moreover, we compute pressure gradients along the surface by first smoothing these pressure along the surface using splines implemented in S_CI_P_Y_ [68,69].

### 2.2. Optimal Sensor Placement Based on Information Gain

In the present work, a swimmer is equipped with sensors that are used to identify the size and location of a nearby school. The optimal sensor locations are identified using Bayesian experimental design [70] so that the information obtained from the collected measurements is maximized. We define the information gain as the distance between the prior belief on the quantities of interest and the posterior belief after obtaining the measurements. Here, we choose as measure of the distance the Kullback–Leibler divergence between the prior and the posterior distribution.

#### 2.2.1. Bayesian Estimation of Swimmers

**Figure 2:**
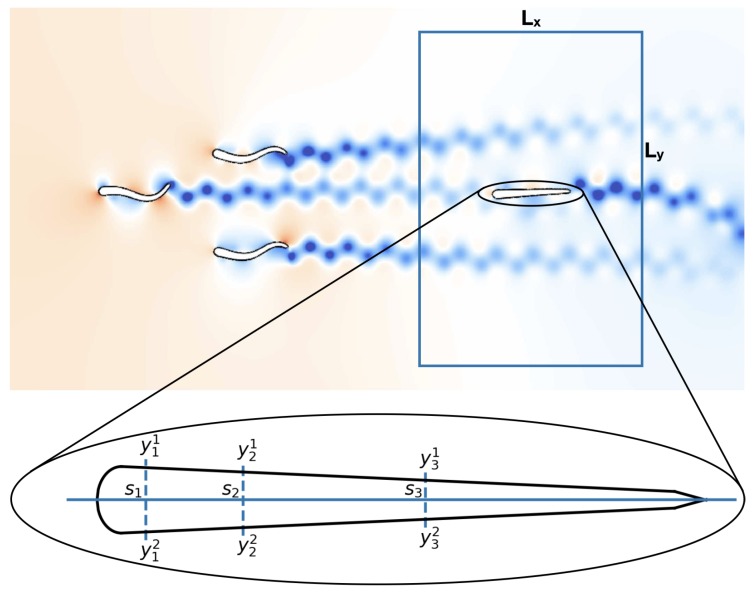
Simulation setup used for determining the optimal sensor distribution on a fish-like body. The follower is initially located inside the rectangular area. The number of swimmers in the leading group is varied between one and eight. The sensor-placement algorithm attempts to find the arrangement of sensors s that allows the follower to determine with lowest uncertainty the relative position r and the number of swimmers $n_{f}$ in the leading group of swimmers. For each sensor $s_{k}$ the swimmer collects measurements $y_{k}^{1}$ and $y_{k}^{2}$ at locations $x_{1}\left(s_{k}\right)$ and $x_{2}\left(s_{k}\right)$ on the skin, respectively.

In the present experiment setup, we consider a group of swimmers followed by a single swimmer. The follower needs to identify (i) the relative location r of the center of mass and (ii) the population $n_{f}$ of the leading group. We denote with ϑ=r or $\vartheta =n_{f}$ these unknown quantities and allow the follower to update its prior belief p(ϑ) about the leading group of swimmers by collecting measurements on its sensors. These sensors are distributed symmetrically on both sides of the swimmer and are represented by a single point on its mid-line. We denote the *k*-th measurement location at the upper and the lower part with $x_{1}\left(s_{k}\right)$ and $x_{2}\left(s_{k}\right)$, respectively. The corresponding measurements are denoted by $y_{k}^{1}$ and $y_{k}^{2}$, respectively (see Figure 2 for a sketch of the setup).

We denote by $F\left(\vartheta ;s\right)\in R^{2n}$ the output of the flow simulation and include an error term ε to account for inaccuracies such as as numerical errors and imperfections in the sensors. The measurements on the swimmer body can be expressed as,

(8) y=F(ϑ;s)+ε.

We model the error term by a multivariate Gaussian distribution ε∼N(0,Σ(s)) with zero mean and covariance matrix $\Sigma \left(s\right)\in R^{2n\times 2n}$. In this case the likelihood of a measurement is given by,

(9) $$\begin{array}{cc} \; & p\left(y|\vartheta ,s\right)=\frac{1}{\sqrt{{\left(2\pi \right)}^{2n}\det\left(\Sigma \left(s\right)\right)}}\exp\left(-\frac{1}{2}{\left(y-F(\vartheta ;s)\right)}^{⊤}\Sigma^{-1}\left(s\right)\left(y-F(\vartheta ;s)\right)\right)\;. \end{array}$$

The covariance matrix depends on the sensor positions s and we assume that the prediction errors are correlated for measurements on the same side of the swimmer and uncorrelated if they originate from opposite sides. Finally, we assume that the correlation is decaying exponentially with the distance of the measurement locations. The functional form of the resulting covariance matrix is given by,

(10) $$\Sigma_{ij}\left(s\right)=\left\{\begin{array}{cc} \sigma^{2}\exp\left(-\frac{∥x_{1}\left(s_{i}\right)-x_{1}\left(s_{j}\right)∥}{\ell }\right)\;, & \;\mathrm{if}1\leq i,j\leq n\;,\\ \sigma^{2}\exp\left(-\frac{∥x_{2}\left(s_{i-n}\right)-x_{2}\left(s_{j-n}\right)∥}{\ell }\right)\;, & \;\mathrm{if}n<i,j\leq 2n\;,\\ 0\;, & \;\mathrm{otherwise}\;, \end{array}\right.$$

where ℓ>0 is the correlation length and σ is the correlation strength. For all the cases described in this work, the correlation length is set to one tenth of the swimmer length ℓ=0.1L. The correlation strength is set to be two times the average of the signals coming from the simulations,

(11) $$\sigma =\frac{1}{n\;N_{\vartheta }}\sum_{j=1}^{2n}\sum_{i=1}^{N_{\vartheta }}\left|F\left(\vartheta^{\left(i\right)};s_{j}\right)\right|\;,$$

where $\vartheta^{\left(i\right)}$ are samples from the distribution p(ϑ). We remark that the covariance matrix must be symmetric and positive definite. To ensure positive definiteness we have to take special care to the case where we pick a sensor location twice. Notice that when $s_{i}=s_{j}$ for i≠j, a non-diagonal entry equals the diagonal entry and positive definiteness is violated. We handle this case by setting the argument of the exponential in Equation (10) to $10^{-7}$ when $s_{i}=s_{j}$. This form of the correlation error reduces the utility when sensors are placed too close together and prevents excessive clustering of the sensors [71,72].

We wish to identify the locations s yielding the largest information gain about the unknown parameter ϑ of the disturbance. A measure for information gain is defined through the Kullback–Leibler (KL) divergence between the prior belief of the parameter values and the posterior belief, i.e., after measuring the environment. The prior and posterior beliefs are represented through the density functions p(ϑ) and p(ϑ|y,s), respectively. We denote by T the support of p(ϑ). The two densities are connected through Bayes’ theorem,

(12) $$p\left(\vartheta |y,s\right)=\frac{p(y|\vartheta ,s)\;p(\vartheta |s)}{p(y|s)}\;,$$

where p(y|ϑ,s) is the likelihood function defined in Equation (9) and p(y|s) is the normalization constant. We assume that the prior belief on the parameters ϑ does not depend on the sensor locations, p(ϑ|s)≡p(ϑ).

The utility function is defined as [73],

(13) $$u\left(s,y\right):=D_{\mathrm{KL}}\left(p\left(\vartheta |y,s\right)||p\left(\vartheta \right)\right)=\int_{T}\ln\frac{p(\vartheta |y,s)}{p(\vartheta )}\;p\left(\vartheta |y,s\right)\vartheta \;.$$

The expected utility is defined as the average value over all possible measurements,

(14) $$\begin{array}{cc} U(s): & =E_{y|s}\left[u(s,y)\right]=\int_{Y}u\left(s,y\right)\;p\left(y|s\right)dy\\ \; & =\int_{Y}\int_{T}\;\ln\frac{p(\vartheta |y,s)}{p(\vartheta )}\;p\left(\vartheta |y,s\right)d\vartheta \;p\left(y|s\right)dy\;, \end{array}$$

where Y is the domain of all possible measurements. Using Equation (12) the expected utility can be expressed as,

(15) $$\begin{array}{c} U\left(s\right)=\int_{Y}\int_{T}\;\ln\frac{p(y|\vartheta ,s)}{p(y|s)}\;p\left(y|\vartheta ,s\right)\;p\left(\vartheta \right)d\vartheta dy\;. \end{array}$$

#### 2.2.2. Estimated Expected Utility for Continuous Random Variables: School Location

When ϑ=r is a continuous random variable and $\vartheta \in \Omega \subset R^{2}$. The estimator for the expected utility in this case can be obtained by approximating the two integrals by Monte Carlo integration using $N_{\vartheta }$ samples from p(ϑ) and $N_{y}$ samples from p(y|ϑ,s) [70]. The resulting estimator is given by,

(16) $$U\left(s\right)\approx \hat{U}\left(s\right)=\frac{1}{N_{\vartheta }N_{y}}\sum_{j=1}^{N_{y}}\sum_{i=1}^{N_{\vartheta }}\left[\ln p\left(y^{\left(i,j\right)}|\vartheta^{\left(i\right)},s\right)-\ln\left(\frac{1}{N_{\vartheta }}\sum_{k=1}^{N_{\vartheta }}p\left(y^{\left(i,j\right)}|\vartheta^{\left(k\right)},s\right)\right)\right]\;,$$

where $\vartheta^{\left(i\right)}∼p_{\vartheta }\left(\cdot \right)$ for $i=1,\ldots ,N_{\vartheta }$ and $y^{\left(i,j\right)}∼p_{y}\left(\cdot |\vartheta^{\left(i\right)},s\right)$ for $j=1,\ldots ,N_{y}$. We remark that the computational complexity of this procedure is mainly determined by the number of Navier–Stokes simulations $N_{\vartheta }$. There is no additional computational burden to compute the $N_{y}$ samples following the measurement error model in Equation (8).

#### 2.2.3. Estimated Expected Utility for Discrete Random Variables: School Size

When ϑ is a discrete random variable with finite support taking values in the set $\left\{\vartheta_{1},\cdots ,\vartheta_{N_{\vartheta }}\right\}$ the expected utility in Equation (15) is given by,

(17) $$\begin{array}{c} U\left(s\right)=\sum_{i=1}^{N_{\vartheta }}p\left(\vartheta_{i}\right)\int_{Y}\;\ln\frac{p(y|\vartheta_{i},s)}{p(y|s)}\;p\left(y|\vartheta_{i},s\right)\;dy\;. \end{array}$$

Here, $\vartheta =n_{f}$ represents the number of swimmers in the leading group. An estimator of the given utility can be obtained by Monte Carlo integration using $N_{y}$ samples from the likelihood distribution $p(y|\vartheta_{i},s)$. The estimator is given by

(18) $$U\left(s\right)\approx \hat{U}\left(s\right)=\frac{1}{N_{y}}\sum_{j=1}^{N_{y}}\sum_{i=1}^{N_{\vartheta }}\;p\left(\vartheta_{i}\right)\;\left[\ln p\left(y^{\left(i,j\right)}|\vartheta_{i},s\right)-\ln\left(\sum_{k=1}^{N_{\vartheta }}p\left(\vartheta_{k}\right)p\left(y^{\left(i,j\right)}|\vartheta_{k},s\right)\right)\right]\;.$$

where $y^{\left(i,j\right)}∼p_{y}\left(\cdot |\vartheta^{\left(i\right)},s\right)$ for $j=1,\ldots ,N_{y}$. Let φ be the random variable representing one of the group configurations. Each group configuration is associated with a unique number $\varphi_{i,\ell }$ for $\ell =1,\ldots ,n_{i}$, where $n_{i}$ is the total number of configurations containing *i* swimmers. With this notation, φ takes values in the set $\left\{\varphi_{i,\ell }\;|\;i=1,\ldots ,8\;,\ell =1,\ldots ,n_{i}\right\}$. For examples of different configurations see Appendix A.

Using the fact that for $i=1,\ldots ,N_{\vartheta }$,

$$p(y,\varphi =\varphi_{k,\ell }|\vartheta =\vartheta_{i},s)=0,\;\;\mathrm{for}\;k\neq i\;,$$

and

$$p\left(y|\vartheta =\vartheta_{i},\varphi =\varphi_{i,\ell },s\right)=p\left(y|\varphi =\varphi_{i,\ell },s\right),\;\;\mathrm{for}\;\ell =1,\ldots ,n_{i}\;,$$

and the assumption

$$p\left(\varphi =\varphi_{i,\ell }|\vartheta =\vartheta_{i},s\right)=\frac{1}{n_{i}},\;\;\mathrm{for}\;\ell =1,\ldots ,n_{i}\;,$$

the likelihood function can be written as,

(19) $$\begin{array}{cc} p(y|\vartheta =\vartheta_{i},s)\; & =\sum_{k=1}^{N_{\vartheta }}\sum_{\ell =1}^{n_{i}}p\left(y,\varphi =\varphi_{k,\ell }|\vartheta =\vartheta_{i},s\right)\\ \; & =\sum_{\ell =1}^{n_{i}}p\left(y,\varphi =\varphi_{i,\ell }|\vartheta =\vartheta_{i},s\right)\\ \; & =\sum_{\ell =1}^{n_{i}}p\left(y|\vartheta =\vartheta_{i},\varphi =\varphi_{i,\ell },s\right)\;p\left(\varphi =\varphi_{i,\ell }|\vartheta =\vartheta_{i}\right)\\ \; & =\frac{1}{n_{i}}\sum_{\ell =1}^{n_{i}}p\left(y|\varphi =\varphi_{i,\ell },s\right)\;. \end{array}$$

Notice that the likelihood function for fixed $\vartheta_{i}$, is a mixture of Gaussian distributions with equal weights and that $p\left(y|\varphi =\varphi_{i,\ell },s\right)=N\left(y|F\left(\varphi_{i,\ell };s\right),\Sigma \left(s\right)\right)$. In order to draw a sample from the likelihood, first we draw an integer $\ell^{*}$ with equal probability from 1 to $n_{i}$ and then draw $y∼p_{y}\left(\cdot |\varphi_{i,\ell^{*}},s\right)$.

The final form of the estimator is given by

(20) $$\begin{array}{c} \hat{U}\left(s\right)=\frac{1}{N_{y}}\sum_{j=1}^{N_{y}}\sum_{i=1}^{N_{\vartheta }}\;p\left(\vartheta_{i}\right)\;\left[\ln\frac{1}{n_{i}}\sum_{\ell =1}^{n_{i}}p\left(y^{\left(i,j\right)}|\varphi_{i,\ell },s\right)-\ln\left(\frac{1}{n_{i}}\sum_{k=1}^{N_{\vartheta }}p\left(\vartheta^{\left(k\right)}\right)\sum_{\ell =1}^{n_{i}}p\left(y^{\left(i,j\right)}|\varphi_{i,\ell },s\right)\right)\right]\;. \end{array}$$

#### 2.2.4. Optimization of the Expected Utility Function

In order to determine the optimal sensor arrangement we maximize the utility estimator $\hat{U}\left(s\right)$ described in Equation (16). It has been observed that the expected utility for many sensors often exhibit many local optima [71,74]. Heuristic approaches, such as the sequential sensor placement algorithm described by [75], have been demonstrated to be effective alternatives. Here, following [75], we perform the optimization iteratively, placing one sensor after the other. We start by placing one sensor $s_{1}^{☆}$ by a grid search in the interval [0,L], where *L* is the length of the swimmer. In the next step we compute the location of the second sensor by setting $s=(s_{1}^{☆},s)$ and repeating the grid search for the new optimal location $s_{2}^{☆}$. This procedure is then continued by defining

(21) $$s_{i}^{☆}=\underset{s}{\mathrm{argmax}}\;\hat{U}\left(s\right)\;\mathrm{where}\;s=\left(s_{1}^{☆},\ldots ,s_{i-1}^{☆},s\right)\;.$$

We note that the scalar variable *s* denotes the mid-line coordinate of a single sensor-pair, whereas the vector s holds the mid-line coordinates of all sensor-pairs. Besides the mentioned advantages, sequential placement allows to quantify the importance of each sensor placed and provides further insight into the resulting distribution of sensors.

## 3. Results

We examine the optimal arrangement of pressure gradient and shear stress sensors on the surface of a swimmer trailing a school of self-propelled swimmers. We consider two sensing objectives: (a) the size of the leading school and (b) the relative position of the school. The simulations correspond to a Reynolds number Re=L2ν=2000. In all experiments, we use 4096 points to discretize the horizontal direction x∈[0,1] and all artificial swimmers have a length of L=0.1.

For the “size of the leading school” experiment, where the aim is to determine the size of the group, we chose the school-sizes to be $\vartheta_{i}=1,\cdots ,8$. First we consider one configuration per group-size. In this case inferring the configuration is equivalent to inferring the number of swimmer in the group. To increase the difficulty we consider $n_{i}$ different initial configurations. In each configuration we assign a number $\varphi_{i,\ell }$ for i=1,⋯,8 and $\ell =1,\cdots ,n_{i}$. In total, we consider $N_{tot}=\sum_{i}n_{i}=61$ distinct configurations each having the same prior probability $1/N_{tot}$. In Appendix A we present the initial condition for all configurations. The center of mass of the school is located at x=0.3 and in the y-axis in the middle of the vertical extent of the domain. We use a controller to fix the distance between *x* and *y* coordinates of two swimmers to Δx=Δy=0.15, see Section 2.1.1.

**Figure 3:**
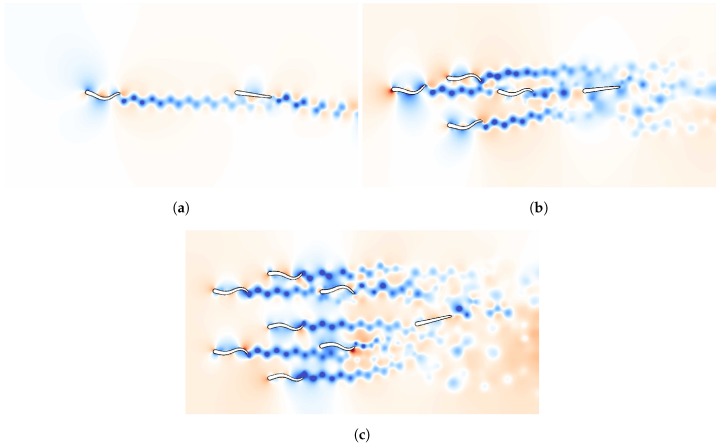
Snapshots of the pressure field in the environment of the follower swimmer generated by one (**a**), four (**b**) and seven (**c**) schooling swimmers. The snapshots are taken at the moment the measurement was performed for one particular location of the follower in the prior region. High pressure is shown in red and low pressure in blue.

For the “relative position” experiment, where the aim is to determine the relative location of the follower to the center of mass of the leading group, we consider three independent experiments with one, four and seven leading swimmers. Snapshots of the pressure field for these simulations are presented in Figure 3. The prior probability for the position of the group is uniform in the domain [0.6,0.8]×[0.1,0.4]. The support of the prior probability is discretized with 21×31 gridpoints. Since the experiments are independent, the total expected utility function for the three cases is the sum of the expected utility of each experiment [56].

For both experiments we record the pressure gradient and shear stress on the surface of the swimmer using the methods discussed in Section 2.1.2. The motion of the swimmer introduces disturbances on its own surface. In order to distinguish the self-induced from the environment disturbances we freeze the movement of the following swimmer and set its curvature to zero. The freezing time is selected by evolving the simulation until the wakes of the leading group are sufficiently mixed and passed the following swimmer. We found that this is the case for T=22. The transition from swimming to coasting motion takes place during the time interval [T,T+1]. Finally, we record the pressure gradient and the shear stress at time T+2. The resulting sensor-signal associated to the midline coordinates s for a given configuration ϑ is denoted F(ϑ;s), see Equation (8).

### 3.1. Utility Function for the First Sensor

**Figure 4:**
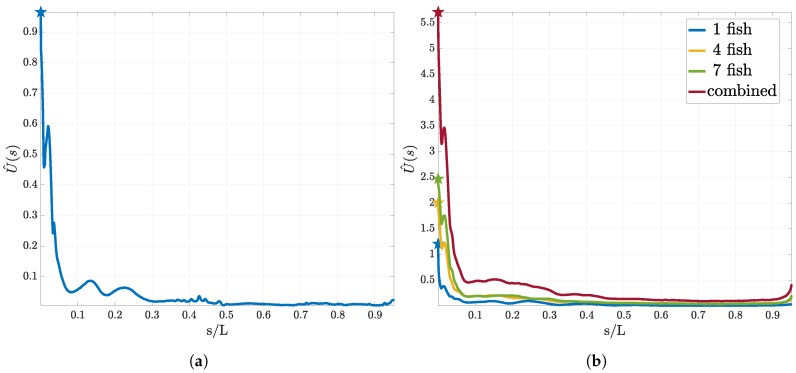
Utility curves for the first sensor using pressure measurements. In (**a**) the utility estimator for the “size of the leading school” experiment is presented. (**b**) corresponds to the utility estimator for the “relative position” experiment. We show the resulting curves for one, three and seven swimmer in the leading group and the total expected utility. We observe that although the form does not drastically change, the total utility increases with increasing size of the leading group.

In this section we discuss the optimal location of a single pressure gradient sensor using the estimators in Equations (16) and (20). Recall that we estimate the expected KL divergence between the prior and the posterior distribution for different sensor locations s. The KL divergence can be understood as a measure of distance between two probability distributions. Thus, higher values of divergence correspond to preferable locations for the sensor, leading to higher information gain. The resulting utilities are plotted in Figure 4. For all experiments we find that the tip of the head (s=0) exhibits the largest utility independent of the number of swimmer in the leading group.

At the tip of the head, the two symmetrically placed sensors have the smallest distance. In Equation (10) we have assumed that the two swimmer halves are symmetric and uncorrelated. Due to the small distance of the sensors at the head, spatial correlation between the sensors across the swimmer halves would decrease the utility of this location. In order to test whether the utility for sensors at the head is influenced by this symmetry assumption, we perform experiments where we place a single sensor on one side of the swimmer. Again, in this case the location at the head is found to have the highest expected utility.

There is evidence that the head experiences the largest variance of pressure gradients F(ϑ;s). The same observations can be made for the density of the sub-canal neuromasts, which is also highest in the front of the fish [47]. To check the presence of this correlation in our study, we examine the variance of the values obtained from our numerical solution of the Navier–Stokes equation. We confirm that our simulations are consistent with this experimental observation. We find that independent of the number of swimmers, the variance in the sensor signal ${\mathrm{var}}_{\vartheta }\left(F\left(\vartheta ;s\right)\right)$ is largest at s=0.

### 3.2. Sequential Sensor Placement

**Figure 5:**
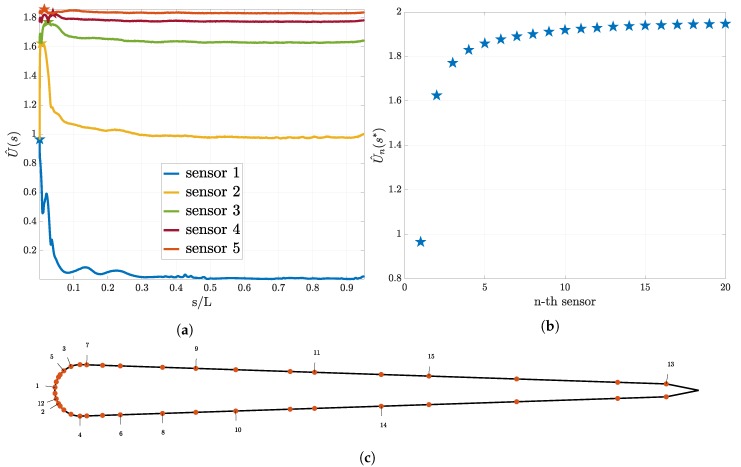
Optimal sensor placement for the pressure sensors and the “size of the leading school” experiment. In (**a**) the utility estimator for the first five sensors and in (**b**) the value of the utility estimator at the optimal sensor location for the first 20 sensors are presented. In (**c**), the distribution of the sensors on the swimmer surface is presented. Here, the numbers associated to each sensor indicate that this location is the *i*-th sensor location chosen according to Equation (21).

In this section we discuss the results of the sequential sensor placement described in Section 2.2.4. For the “size of the leading school” experiment we present the results in Figure 5. In Figure 5a the utility curve for the first five sensors is shown. We observe that the utility curve becomes flatter as the number of sensors increase. Furthermore, we observe that the location where the previous sensor was placed is a minimum for the utility for the next sensor. Figure 5b shows the utility estimator at the optimal sensor for up to 20 sensors and it is evident that the value of the expected utility reaches a plateau. In Figure 5c the found optimal location of the sensors on the skin of the swimmer is presented. The numbers correspond to the iteration in the sequential procedure that the sensor was placed. Note that the sensors are being placed symmetrically.

**Figure 6:**
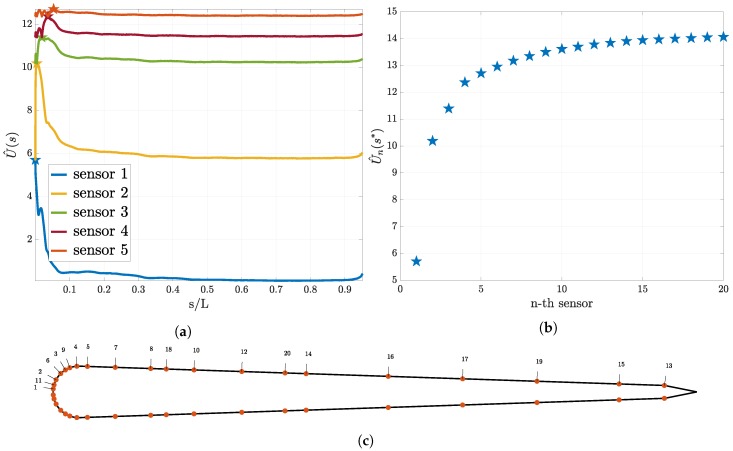
Optimal sensor placement for the pressure gradient sensors for the “relative position” experiment. In (**a**), the utility estimator for the first five sensors and in (**b**) the value of the utility estimator at the optimal sensor location for the first 20 sensors are presented. In (**c**), the distribution of the sensors on the swimmer surface is presented. Here, the numbers associated to each sensor indicate that this location is the *i*-th sensor location chosen according to Equation (21).

The optimal sensor placement results for the “relative position” experiment can be found in Figure 6. Similar to the other experiment the utility curves become flatter after every placed sensor and the location for the previous sensor is a minimum for the utility for the next sensor (see Figure 6a). We plot the maximum of the utility for up to 20 sensors (see Figure 6b) and observe a convergence to a constant value. In Figure 6c the found optimal location of the first 20 sensors is presented.

For both experiments, it is evident that the utility of the optimal sensor location approaches a constant value. This fact can be explained by recalling that the expected utility in Equation (15) is a measure of the averaged distance between the prior and the posterior distribution. Increasing the number of sensors leads to an increase in the number of measurements. By the Bayesian central limit theorem, increasing the number of measurements leads to convergence of the posterior to a Dirac distribution. As soon as the posterior has converged, the expected distance from the prior, and thus the expected utility, remains constant.

The found sensor distributions for the two objectives are similar, having clusters at the head and uniform distribution along the body. In order to underpin the biological relevance of the observed sensor distribution we compare our results to [47]. Given that the canals display significant 3D branching in the head a direct comparison is difficult. However, the found cluster of sensors at the head agrees qualitatively with the high canal density reported in [47].

### 3.3. Inference of the Environment

In this section we demonstrate the importance of the optimal sensor locations and examine the convergence of the posterior distribution. We compute the posterior distribution via Bayes’ theorem given in Equation (12). We set y=F(ϑ,s) and compute the posterior for different values of ϑ in the prior region. We consider measurements collected at: (a) the optimal and (b) the worst sensors location.

**Figure 7:**
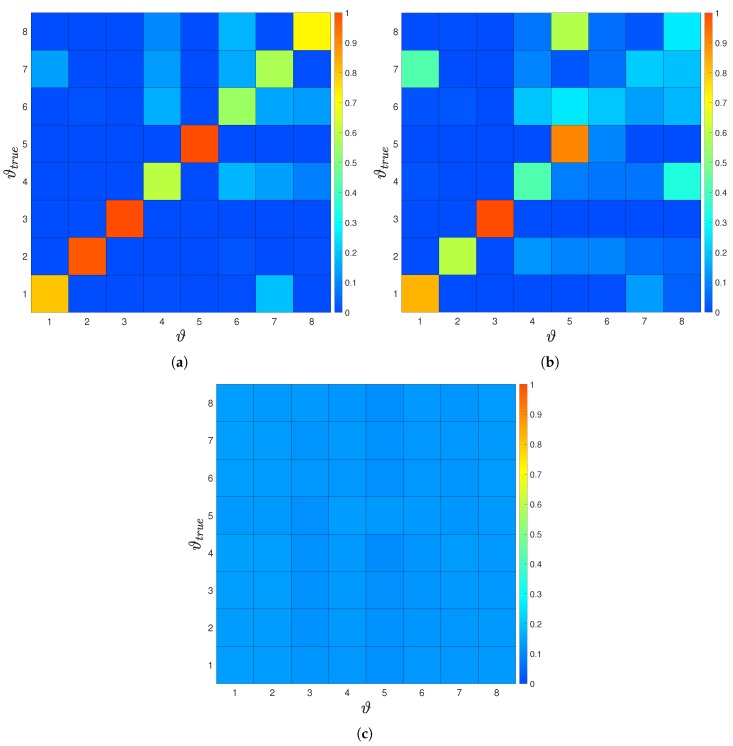
(**a**) Estimated posterior probability for a single sensor optimally placed and a single configuration per group size. The posterior shows clear peaks at the correct number of swimmer for all cases, leading to perfect inference of the parameter of interest. The posterior probability for (**b**) optimal and (**c**) worst sensor location for multiple configurations per group size. Here, for the optimal sensor location and one, two, three and five swimmer we see a clear peak for the true size of the group. For the worst sensor location the posterior is almost uniform and does not allow to extract any information about the size of group.

The posterior probability for the “size of the leading school” experiment is shown in Figure 7. We observe that the worst sensor location implies an almost uniform posterior distribution, reflecting that measurements at this sensor carry no information. On the other hand, the posterior distribution for the optimal sensor is more informative. We observe that for groups with small size the follower is able to identify the size with more confidence, as opposed to larger groups. We compare the posterior for an experiment with only one configuration per group-size to an experiment with multiple configurations. For multiple configurations the posterior is less informative. This indicates that the second case occurs to be a more difficult problem. Finally, notice that the posterior for one configuration is symmetric, where when adding multiple configurations this symmetry is broken. This fact is discussed in Appendix B.

**Figure 8:**
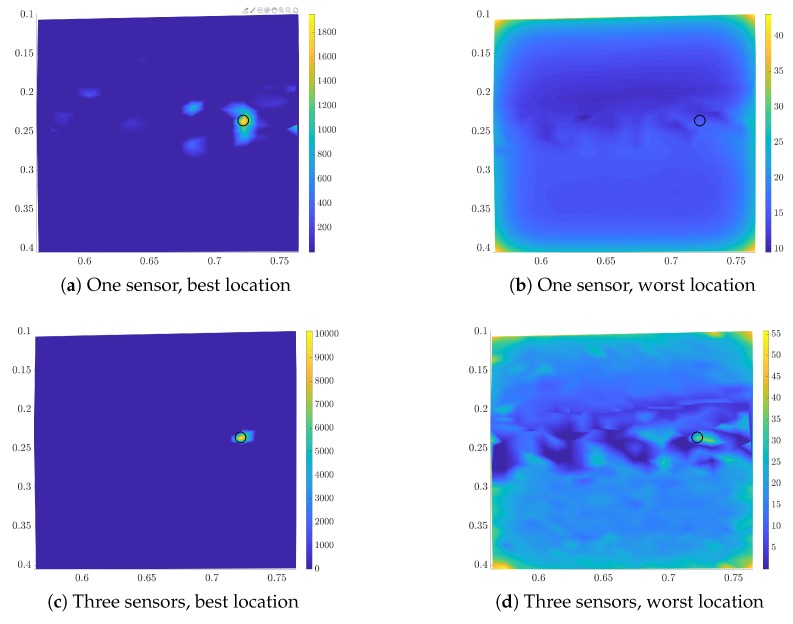
Estimated posterior for the final location for the best (left column) and worst (right column) sensor-location for one (upper row) and three sensors (lower row). Light colors correspond to high probability density values. We marked the actual location with a black circle.

The posterior density for the “relative position” with one leading swimmer is presented in Figure 8. The posterior for the configuration with three and seven swimmers is similar. We compute the posterior for measurements at the best and the worst location for one and three sensors. For the three sensors the worst location has been selected in all three phases of the sequential placement. The results for the normalized densities are shown in Figure 8. We observe that one sensor at the optimal location gives a very peaked posterior. Three optimal sensors can infer the location with low uncertainty. This is not the case for the worst sensors, where adding more sensors does not immediately lead to uncertainty reduction.

### 3.4. Shear Stress Sensors

In this section, we discuss the results for the optimal positioning of shear stress sensors. We follow the same procedure as in Section 3.1 and Section 3.2. Here, we omit the presentation of all the results and focus on the similarities and differences to the pressure gradient sensors.

The optimal location for a single sensor for the “size of the leading school” experiment is at $s^{*}=3.01\times 10^{-4}$. For the “relative position” experiment we find the optimal location $s^{*}=3.84\times 10^{-4}$. In contrast to the optimal location for one pressure gradient sensor, the found sensor is not at the tip of the head and is at different positions for the two experiments. Examining the variance in the shear signal shows quantitatively the same behaviour as the utility. Comparing the location of the maxima in variance shows that they do not coincide with the found maxima for the expected utility for shear sensors.

**Figure 9:**
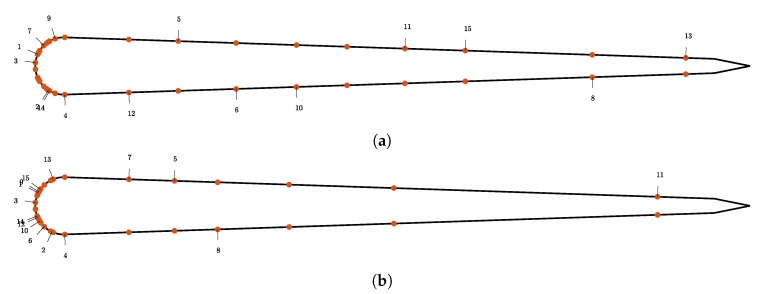
Optimal sensor locations for the shear stress measurements for the “size of the leading school” in (**a**) experiment and “relative position” experiment in (**b**).

We perform sequential placement of 15 sensors. The resulting distribution of sensors is shown in Figure 9. In Section 3.2 we argue that the expected utility must reach a plateau when placing many sensors using the Bayesian central limit theorem. For shear stress sensors we observe that the convergence is slower compared to the pressure gradient sensors. We conclude that the information gain per shear stress sensor placed is lower as for the pressure gradient sensors.

The posterior density obtained for both experiments is less informative when using the same number of sensors. Also this indicates that shear is a less informative quantity yielding a slower convergence of the posterior. This is in agreement with the observation that the subcanal neuromasts associated with pressure gradient sensing are more robust to noise [28]. For multiple fish in schools the resulting flow field is disturbed, thus suggesting the use of pressure gradient sensors.

## 4. Discussion

We present a study of the optimal sensor locations on a self-propelled swimmer for detecting the size and location of a leading group of swimmers. This optimization combines Bayesian experimental design with large scale simulations of the two dimensional Navier–Stokes equations. Mimicking the function of sensory organs in real fish, we used the shear stress and pressure gradient on the surface of the swimmers to determine the sensor feedback generated by a disturbance in the flow field.

The optimization was performed for different configurations of swimmers, ranging from a simple leader-follower configuration with two swimmers, to a group of up to eight swimmers leading a single follower. We considered two types of information: the number of swimmers in the leading group and the relative location of the leading group. We find that, although the general shape of the utility function varies between the two objectives, the preferred location of the first sensor on the head of the swimmer is consistent. Furthermore, we find that the objective is only weakly influenced when varying the number of members in the leading group.

We perform a sequential sensor placement and find that the utility converges to a constant value and thus we can conclude that few sensors suffice to infer the quantities of the surrounding flow. Indeed, we find that the optimal sensor locations correspond to a posterior distribution that is strongly peaked around the true value of the quantity of interest. In summary, we find, that for the group sizes under examination, changing the number of swimmers in the leading group does not influence the follower’s ability to infer the mean school location. Furthermore, we were able to show that choosing the locations for the measurements in a systematic way we are able to infer the number of swimmer in the leading group and the location of our agent to high accuracy.

We envision that the presented methodology can provide guidance in developing autonomous systems of schooling artificial swimmers. While biological organisms have distinct flow fields from those examined in the present two-dimensional simulations, we believe that the algorithms presented here in can be extended to 3D flows. Moreover, while we draw a distinction between fish and the studied artificial swimmers, we note the capability of identifying neighboring swimmers using shear and pressure information on the body of the swimmers, indicating sufficiency of such type of information for flow sensing.

## Figures and Tables

**Figure A1:**
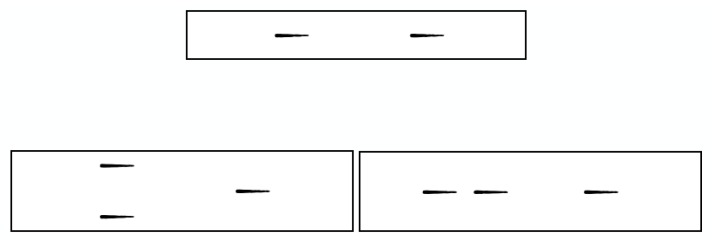
Configurations for two leading swimmers.

**Figure A2:**
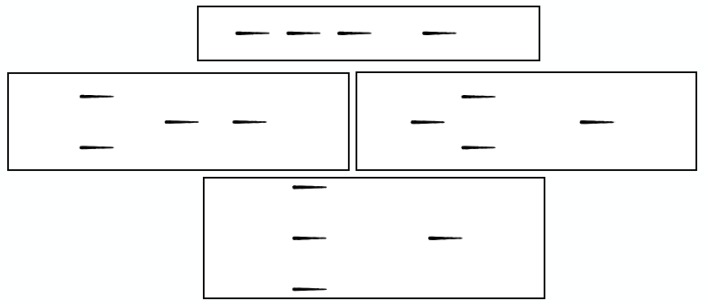
Configurations for three leading swimmers.

**Figure A3:**
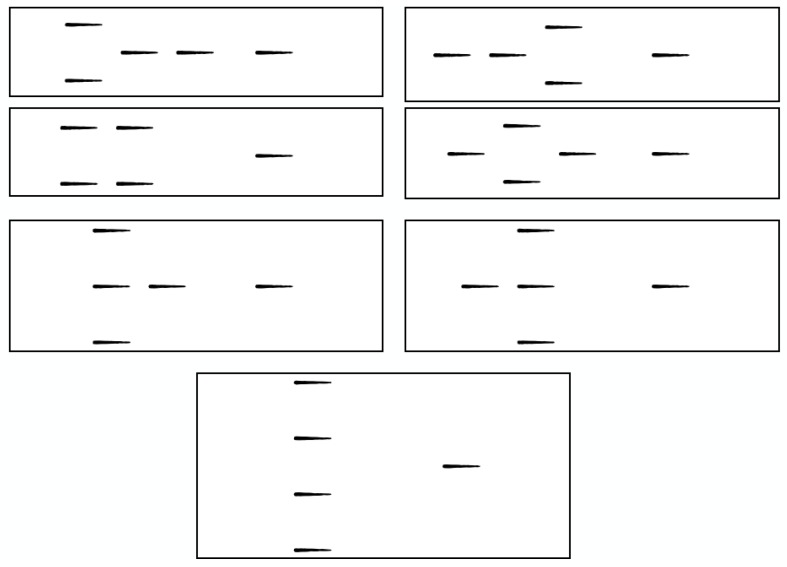
Configurations for four leading swimmers.

**Figure A4:**
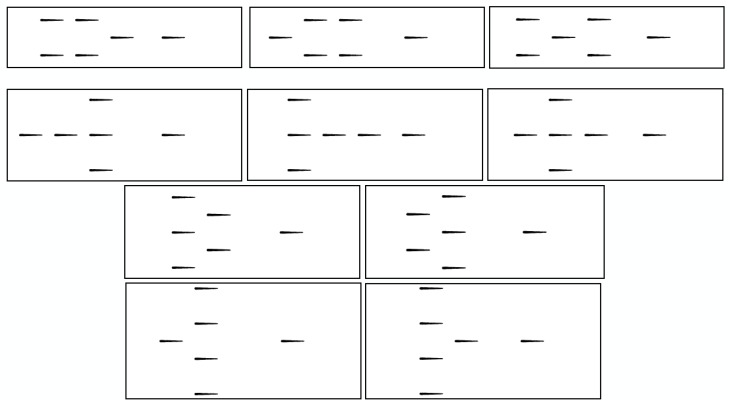
Configurations for five leading swimmers.

**Figure A5:**
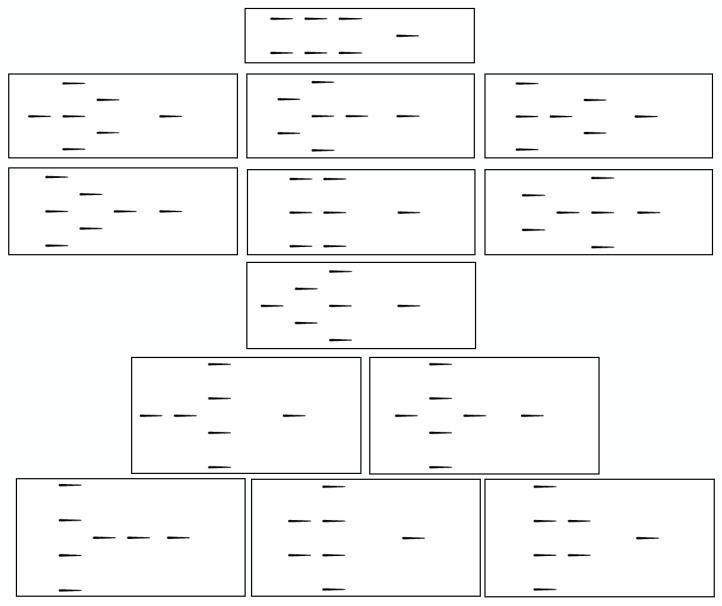
Configurations for six leading swimmers.

**Figure A6:**
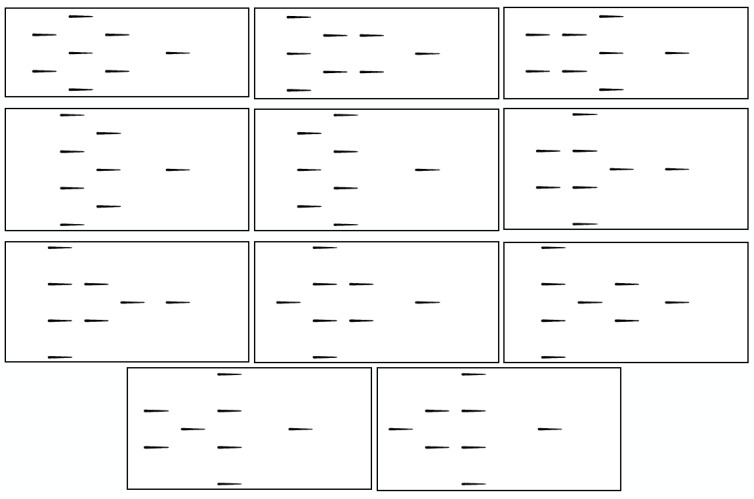
Configurations for seven leading swimmers.

**Figure A7:**
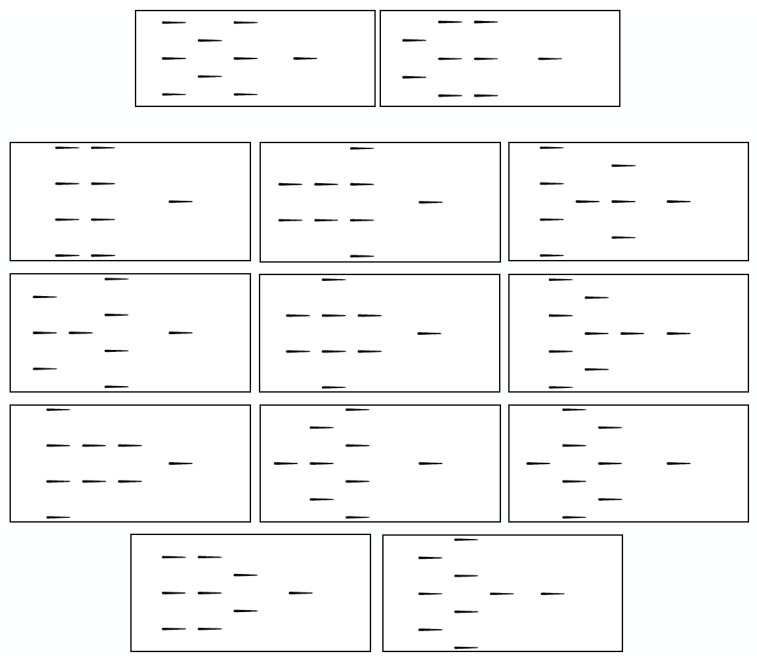
Configurations for eight leading swimmers.

## Appendix A. Configurations

The configuration used for the “size of the leading school” experiment. For the configurations with three rows the vertical extent y∈[0,0.5] was discretized using 2048 gridpoints, for the ones with four rows it was extended to y∈[0,0.75] and discretized using 3072 gridpoints.

## Appendix B. The Posterior Is Not Symmetric

The estimated posterior in Figure 7b is not symmetric with respect to the $\vartheta_{\mathrm{true}}=\vartheta$ diagonal. This observation indicates that the posterior is not symmetric with respect to an exchange of ϑ and $\vartheta_{\mathrm{true}}$, the parameter we try to infer and the one used in the simulation. Here, we want to show that this observation is true in general. In order to lighten the notation, we neglect the dependence of the distributions on the sensor location s.

In Section 2.2.3 we showed that the distribution of $\vartheta_{i}$ conditioned on measurements y, under the assumption of uniform prior, is proportional to

(A1) $$p\left(\vartheta_{i}|y\right)\propto p\left(y|\vartheta_{i}\right)\;p\left(\vartheta_{i}\right)=\frac{1}{8}\frac{1}{n_{i}}\sum_{\ell =1}^{n_{i}}\frac{1}{\sqrt{2\pi \sigma^{2}}}\exp\left(-\frac{{\left(y-F\left(\varphi_{i,\ell }\right)\right)}^{2}}{2\sigma^{2}}\right)\;.$$

We want to show that for any i≠j,

(A2) $$p\left(\vartheta_{i}|\vartheta_{j}\right)=p\left(\vartheta_{i}|y=F\left(\varphi_{j,k}\right)\right)\neq p\left(\vartheta_{j}|y=F\left(\varphi_{i,\ell }\right)\right)=p\left(\vartheta_{j}|\vartheta_{i}\right)\;,$$

for any configurations $\varphi_{j,k}$ and $\varphi_{i,\ell }$ corresponding to a school of size $\vartheta_{j}$ and $\vartheta_{i}$, respectively. From Equation (A1) it is easy to see that (A2) is true due to the fact that

(A3) $$\frac{1}{n_{i}}\sum_{\ell^{\prime }=1}^{n_{i}}\exp\left(-\frac{{\left(F\left(\varphi_{j,k}\right)-F\left(\varphi_{i,\ell^{\prime }}\right)\right)}^{2}}{2\sigma^{2}}\right)\neq \frac{1}{n_{j}}\sum_{k^{\prime }=1}^{n_{j}}\exp\left(-\frac{{\left(F\left(\varphi_{i,\ell }\right)-F\left(\varphi_{j,k^{\prime }}\right)\right)}^{2}}{2\sigma^{2}}\right)\;.$$

Finally, we note that in the case where we have only one configuration per group size, i.e., $n_{i}=1$ for all *i*, the statement in (A3) is not true and the posterior is symmetric.
